# Process development for the production of 15β-hydroxycyproterone acetate using *Bacillus megaterium* expressing CYP106A2 as whole-cell biocatalyst

**DOI:** 10.1186/s12934-015-0210-z

**Published:** 2015-03-05

**Authors:** Flora M Kiss, Marie T Lundemo, Josef Zapp, John M Woodley, Rita Bernhardt

**Affiliations:** Institute of Biochemistry, University of Saarland, Campus B 2.2, 66123 Saarbruecken, Germany; Institute of Pharmaceutical Biology, University of Saarland, Campus B 2.2, 66123 Saarbruecken, Germany; CAPEC-PROCESS, Department of Chemical and Biochemical Engineering, Technical University of Denmark, DK-2800 Lyngby, Denmark

**Keywords:** Cytochrome P450, CYP106A2, *Bacillus megaterium*, Whole-cell biocatalyst, Steroid hydroxylase, Cyproterone acetate, Bioreactor, Cyclodextrin

## Abstract

**Background:**

CYP106A2 from *Bacillus megaterium* ATCC 13368 was first identified as a regio- and stereoselective 15β-hydroxylase of 3-oxo-∆^4^-steroids. Recently, it was shown that besides 3-oxo-∆^4^-steroids, 3-hydroxy-∆^5^-steroids as well as di- and triterpenes can also serve as substrates for this biocatalyst. It is highly selective towards the 15β position, but the 6β, 7α/β, 9α, 11α and 15α positions have also been described as targets for hydroxylation. Based on the broad substrate spectrum and hydroxylating capacity, it is an excellent candidate for the production of human drug metabolites and drug precursors.

**Results:**

In this work, we demonstrate the conversion of a synthetic testosterone derivative, cyproterone acetate, by CYP106A2 under *in vitro* and *in vivo* conditions. Using a *Bacillus megaterium* whole-cell system overexpressing CYP106A2, sufficient amounts of product for structure elucidation by nuclear magnetic resonance spectroscopy were obtained. The product was characterized as 15β-hydroxycyproterone acetate, the main human metabolite. Since the product is of pharmaceutical interest, our aim was to intensify the process by increasing the substrate concentration and to scale-up the reaction from shake flasks to bioreactors to demonstrate an efficient, yet green and cost-effective production. Using a bench-top bioreactor and the recombinant *Bacillus megaterium* system, both a fermentation and a transformation process were successfully implemented. To improve the yield and product titers for future industrial application, the main bottlenecks of the reaction were addressed. Using 2-hydroxypropyl-β-cyclodextrin, an effective bioconversion of 98% was achieved using 1 mM substrate concentration, corresponding to a product formation of 0.43 g/L, at a 400 mL scale.

**Conclusions:**

Here we describe the successful scale-up of cyproterone acetate conversion from shake flasks to bioreactors, using the CYP106A2 enzyme in a whole-cell system. The substrate was converted to its main human metabolite, 15β-hydroxycyproterone acetate, a highly interesting drug candidate, due to its retained antiandrogen activity but significantly lower progestogen properties than the mother compound. Optimization of the process led to an improvement from 55% to 98% overall conversion, with a product formation of 0.43 g/L, approaching industrial process requirements and a future large-scale application.

## Background

Cytochrome P450 monooxygenases (P450s) are hemeproteins that catalyze the oxidation of a wide variety of substrates, from endogenous compounds, such as steroids, vitamins and fatty acids, to xenobiotics and drugs. They have attracted interest in research due to their unique ability to activate molecular oxygen, introducing one atom into the substrate and reducing the second one to water [1]. The fact that they are able to introduce an oxygen atom into non-activated hydrocarbons without extreme conditions, in combination with their high regio- and stereoselectivity on complex molecules, makes their application particularly interesting to the pharmaceutical industry. P450s represent a suitable alternative over chemical synthesis, especially in the hydroxylation of steroidal pharmaceuticals, where the chemical methods are either time- and labor-intensive, expensive and complex or non-existent [2]. Steroid-based drugs are one of the largest groups of marketed pharmaceuticals [3]. There are about 300 approved steroid drugs to date and their number is constantly growing due to the production of diversely functionalized steroid cores, resulting in often altered therapeutic activity [4]. Thus, steroid-hydroxylating P450s could provide an alternative for the production of drug precursors and human drug metabolites.

The bacterial P450, CYP106A2 from *Bacillus megaterium* (*B. megaterium*) ATCC 13368, is one of the few cloned bacterial steroid hydroxylases that has been studied in detail and was announced to be a suitable biocatalyst for the production of hydroxysteroids [5]. CYP106A2, also known as 15β-hydroxylase, converts mainly 3-oxo-Δ^4^-steroids [6-8] although recent studies have shown that it can perform di- and triterpenoid hydroxylation [9-11] and the conversion of 3-hydroxy-Δ^5^-steroids [12]. Moreover, as a result of on-going screening of a natural substrate library, the substrate range of this enzyme is constantly extended. However, the native substrate of CYP106A2, and thus its biological function, is still unknown. Its natural electron transfer protein is also unknown, yet the activity was successfully demonstrated using megaredoxin and megaredoxin reductase [7] and it is also supported by the bovine adrenal redox partners as well as by putidaredoxin and putidaredoxin reductase [5,13,14].

In the past two decades CYP106A2 was profoundly investigated as a biocatalyst, applying the enzyme in whole-cell systems, efficiently using both *Escherichia coli* (*E. coli*) [5,13] and *B. megaterium* as expression hosts [9-12]. Whole-cell systems, in which the P450 is expressed by a microbial host, have the advantage of stabilizing the enzyme under process conditions and providing cofactor regeneration through cellular metabolism, avoiding the need for the expensive NADPH supply.

Since the transport of hydrophobic substances across the outer membrane of *E. coli* was found to be limiting [5], the attention was shifted to the gram-positive *B. megaterium* as host. This spore-forming, mainly aerobic bacterium became a long-term participant of the biotechnological industry, due to the fact that even the wild-type strains are capable of producing high titers of proteins of industrial interest [15,16]. Further attractive characteristics include the ability to grow on a variety of carbon sources, the GRAS status, high plasmid stability and the lack of endotoxin and extracellular protease production. These characteristics make this organism highly favored for industrial practice [17].

The endogenous CYP106A2 system in *B. megaterium* ATCC 13368 was used for *in vivo* transformation of the diterpene abietic acid producing 12β-hydroxyabietic acid and 12α- hydroxyabietic acid [10]. As a next step, the CYP106A2 gene was overexpressed in combination with bovine adrenal redox partners in *B. megaterium* MS941 for the hydroxylation of 11-keto-β-boswellic acid in the 15α position [9]. Another triterpenoid, dipterocarpol, was also hydroxylated by CYP106A2 in *B. megaterium* ATCC 13368 resulting in six products at a 1 L scale [11]. Recently reported, the regioselective hydroxylation of the 3-hydroxy-Δ^5^ steroid dehydroepiandrosterone (DHEA) was achieved by CYP106A2 expressed in the natural host *B. megaterium* ATCC 13368 and the recombinant *B. megaterium* MS941 [12].

**Scheme 1:**
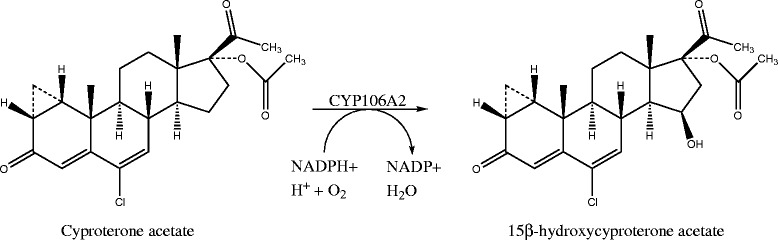
**Cyproterone acetate conversion to its main human metabolite 15β-hydroxycyproterone acetate by CYP106A2.**

In the present work, the conversion of a synthetic testosterone derivative, cyproterone acetate (CPA, 6-Chloro-1β,2β-dihydro-17-hydroxy-3′H-cyclopropa(1,2)-pregna-1,4,6-triene-3,20-dione acetate), was performed using a recombinant *B. megaterium* MS941 system overexpressing the CYP106A2 enzyme. The synthetic antiandrogen, CPA, was converted to 15β-hydroxy cyproterone acetate (15β-OH-CPA, 15β-hydroxy-6-Chloro-1β,2β-dihydro-17-hydroxy-3′H-cyclopropa(1,2)-pregna-1,4,6-triene-3,20-dione acetate) (Scheme 1). CPA is a synthetic derivative of 17α-hydroxyprogesterone, an anti-androgenic compound with additional progestogen and antigonadotropic properties [18,19]. It has antagonistic properties towards the androgen receptor, although it can also act as its partial agonist. It is generally used as a treatment for metastatic prostate cancer and for the control of libido in severe hypersexuality and/or sexual deviation in males, but it is also applied for the treatment of hirsutism and acne in female patients and in oral contraceptive pills. The main human metabolite of CPA in both plasma and urine is the 15β-OH-CPA. It shows only 10% of the progestogenic potency of CPA but retains the anti-androgen activity [19]. These characteristics imply that the metabolite is potentially a better option for the treatment of androgen-induced problems, particularly in male patients. In 1982, the *B. megaterium* ATCC 13368 strain was already proposed for the bioconversion of 1α,2α-methylene steroids into their 15β-hydroxy derivatives, in order to produce new anti-androgenic steroids with minor progestogen activity [20]. According to our latest information, 1 mg 15β-hydroxy metabolite costs 300 $, while 250 mg of the original compound costs 199 $ (Santa Cruz Biotechnology, http://www.scbt.com/, 2014). Although we lack detailed information about the production, the price difference suggests an expensive procedure.

In the present work we perform process development of the CPA bioconversion in shake flasks and lab-scale bioreactors, thus providing an improved model for a greener yet cost-effective large-scale production of the 15β-hydroxy metabolite. The reaction was successfully carried out at 400 mL scale, although to further improve the conversion rate the bottlenecks of the system were identified. Working with P450s applied in whole-cell systems, the following difficulties have already been recognized [1,21]:

- NADP(H) depletion
- low substrate and product solubility
- problematic uptake of the substrate and efflux of the product
- substrate or product inhibition/toxicity.

To find the bottleneck of our system, we addressed each point separately. The cofactor limitation was investigated by adding NADPH in excess. Issues with solubility, toxicity or inhibition, related to substrate or product were investigated. Subsequently, substrate feeding strategies were evaluated in an attempt to overcome these effects. Moreover, the proposed transport restriction was addressed by using different membrane permeabilization methods (freeze-thawing, ultrasonication, acetone treatment). 2-hydroxypropyl-β-cyclodextrin (HP-β-CD) was also applied to improve the process performance since it was previously described to be successful as a solubilizing agent and was used for improved substrate transport across the cell membrane. By identifying the limitations of the system, the aim was to be able to improve the economic performance of the process by increasing the reaction yield at higher substrate concentrations.

## Results

### Purification and spectral characterization of CYP106A2

The CYP106A2 protein was expressed and purified using a recombinant *E. coli* C43 (DE3) strain. The UV–vis absorbance spectra recorded from 250 to 700 nm showed the characteristic absorbance peaks at 356, 417, 534 and 567 nm in the oxidized form. In the case of the reduced and carbon monoxide-bound form, the peak at 450 nm was observed, with no peak indicating inactive P450 at 420 nm.

### *In vitro* conversion, reaction kinetics and inhibition studies

**Figure 1:**
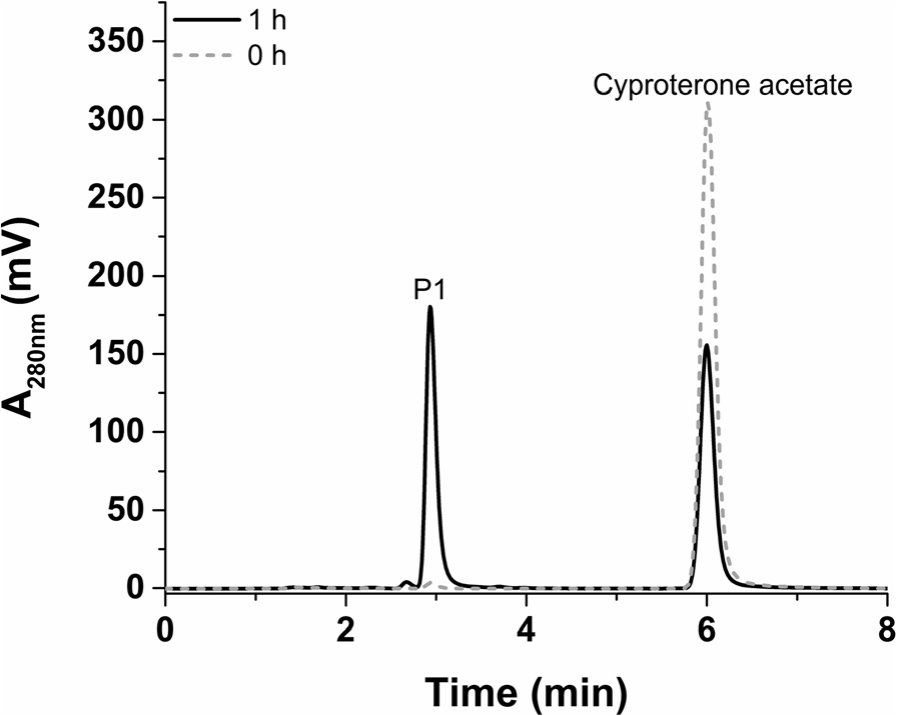
**HPLC chromatogram of the** ***in vitro*** **conversion of cyproterone acetate by CYP106A2.** The reaction was performed in 50 mM potassium phosphate buffer containing 20% glycerol (pH 7.4) at 30°C, using 0.5 μM CYP106A2, 10 μM Adx_4–108_ and 1 μM AdR. The reaction was stopped and extracted twice by ethyl acetate directly after the addition of the substrate (grey dotted line, 0 h) and after 1 h (black line, 1 h). Cyproterone acetate (400 μM) was converted to one main product (P1).

Using difference spectroscopy, the binding of CPA to CYP106A2’s active site was studied *in vitro*. CPA did not induce any spectral shift, indicating that the steroid does not contribute to the replacement of the axial water molecule, hindering the determination of the dissociation constant. The catalytic activity of CYP106A2 towards CPA was first tested *in vitro*. The activity was reconstituted using bovine adrenal redox partners (Adx_4–108_ and AdR) proven to be highly efficient electron suppliers for CYP106A2 [14,22]. The CYP106A2-dependent conversion of CPA was analyzed by high-performance liquid chromatography (HPLC) and resulted in one main product. Using 0.5 μM CYP106A2 and 400 μM substrate, the conversion reached 48.2 ± 2.8% in 60 minutes (Figure 1).

**Figure 2:**
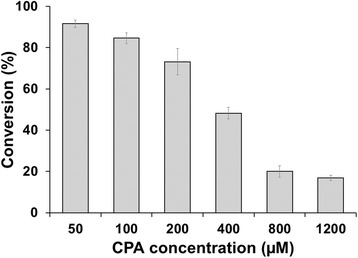
***In vitro*** **conversion of cyproterone acetate using increasing substrate concentrations.** The reaction was performed using substrate concentrations, in a range of 50 μM to 1.2 mM for 60 min. Each bar represents the mean value of three independent measurements, with the corresponding standard deviation indicated by the error bars.

The *in vitro* conversions were also performed with increasing substrate concentrations (50 μM-1.2 mM) to study the potential inhibitory effect of the substrate. Using 200 μM or higher substrate concentrations, the product concentration never exceeded 200 μM. These results suggest that the enzyme is inhibited above a certain product concentration, regardless of the amount of substrate, since the reaction stops after 150 to 200 μM product was formed (Figure 2).

**Figure 3:**
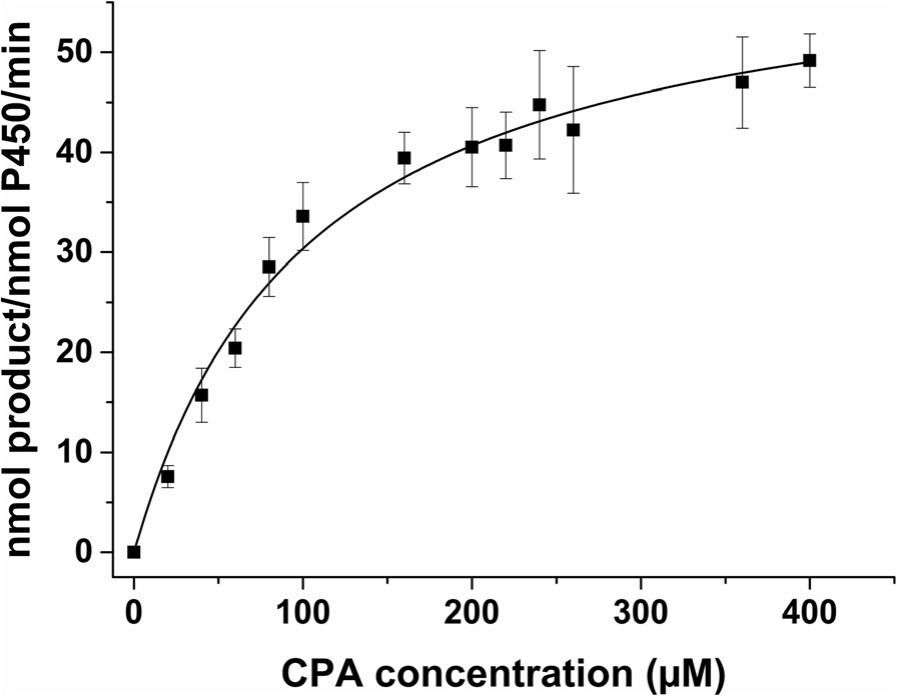
**Determination of the kinetic parameters for the cyproterone acetate conversion catalyzed by CYP106A2.** The reaction kinetics were performed in 50 mM potassium phosphate buffer containing 20% glycerol (pH 7.4) at 30°C for 2 min using 0.5 μM CYP106A2, 10 μM Adx_4–108_ and 1 μM AdR. Cyproterone acetate was used in a concentration range of 0 to 400 μM. The data shown are the result of four independent measurements (R^2^>0.98).

As a next step, the Michaelis-Menten parameters for the CYP106A2-dependent CPA conversion were determined. The catalytic activity of the CPA conversion showed a V_max_ of 61.65 ± 2.56 nmol product per nmol CYP106A2 per minute and a K_m_ of 103.14 ± 11.99 μM (Figure 3).

**Figure 4:**
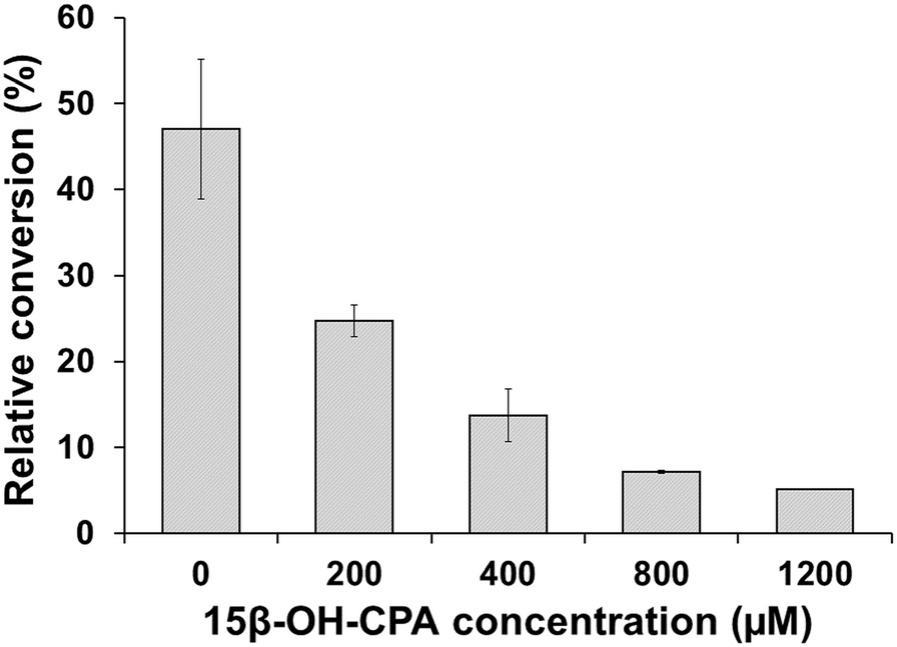
***In vitro*** **conversion of cyproterone acetate using increasing initial product concentrations.** The reactions were performed using 400 μM substrate concentration and initial product concentrations ranging from 0 to 1200 μM for 60 min. The data represents the mean of three independent measurements with the corresponding standard deviation shown by the error bars.

To investigate potential product inhibition, *in vitro* product inhibition experiments were performed using a fixed amount of substrate with increasing initial product concentrations (purified by preparative HPLC). The results confirmed the assumption that the product used in 200 μM or higher concentrations strongly inhibits the reaction. Using 200 μM 15β-OH-CPA, we could observe only a 25 ± 1.9% conversion, which was roughly half of the product formation that was observed in the control sample (47 ± 4.3%) containing only the substrate (400 μM). When increasing the product concentration to 1200 μM the conversion drastically decreased to 5 ± 0.12%, one tenth of the control conversion (Figure 4).

### *In vivo* conversion, product localization and catalyst reusability

**Figure 5:**
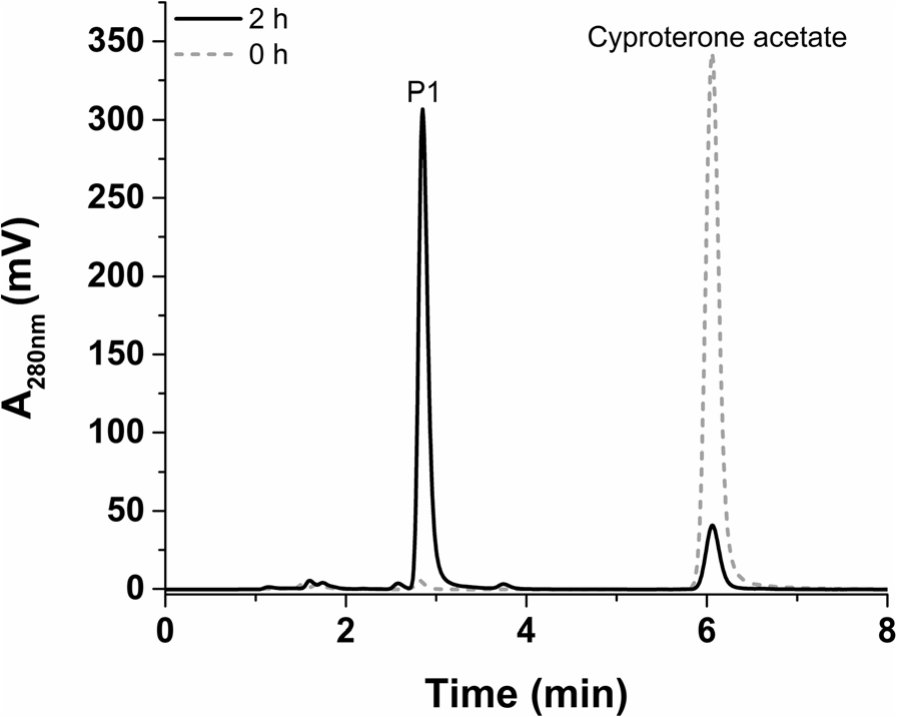
**HPLC chromatogram of the cyproterone acetate conversion using** ***B. megaterium*** **MS941 overexpressing the CYP106A2 enzyme.** The reaction was performed with resting cells in 100 mM potassium phosphate buffer (pH 7.4) at 30°C, 150 rpm. Cyproterone acetate (400 μM) was added to the cells in DMSO solution (2% v/v). Samples were collected directly at the point of substrate addition (grey dotted line) and after 2 h (black line). The substrate was converted to one main product (P1).

**Figure 6:**
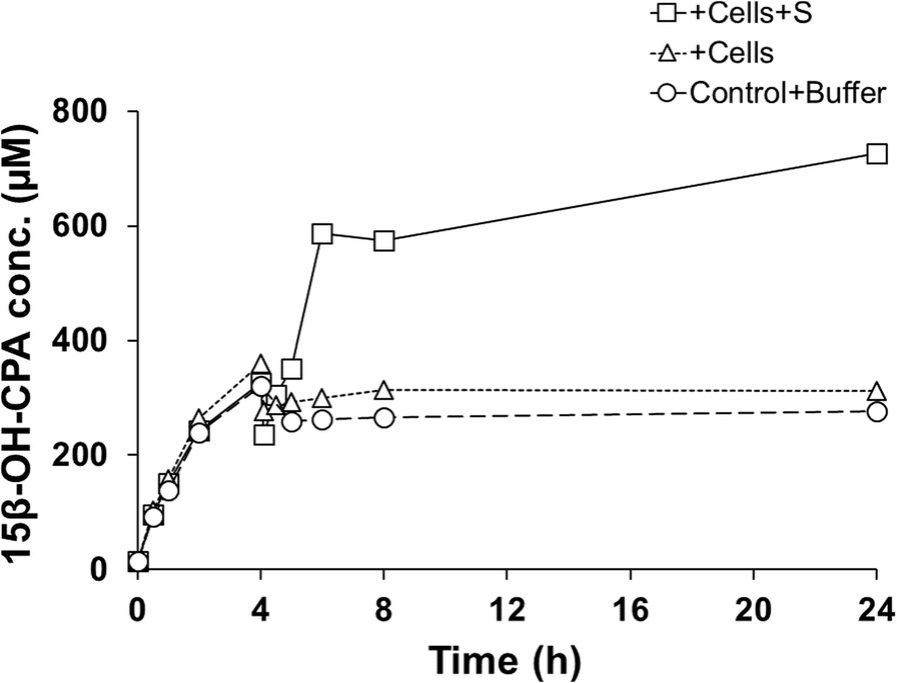
**Effect of whole-cell catalyst addition, with and without additional substrate, on 15**β**-hydroxycyproterone acetate production.** Reactions were performed in shake flasks with 1 mM cyproterone acetate and an initial catalyst concentration of 50 g/L WCW. After 4 h, additional 150 g/L WCW was added to the reaction represented with △, both catalyst and substrate (1 mM) were added to the reaction represented with □ and buffer was added to the control (○) to compensate for the change in volume.

Following the successful conversion of CPA by the CYP106A2-overexpressing *B. megaterium* strain (Figure 5), the product of the reaction was purified on preparative HPLC and its structure was identified by nuclear magnetic resonance (NMR) spectroscopy. The resulting main product (P1), 15β-OH-CPA, was used in the above-mentioned *in vitro* as well as in the *in vivo* product inhibition studies. The whole-cell catalyst was further characterized by examining the substrate and product localization. Both substrate and product were shown to be attached to the cell pellet fraction (data not shown). Adding more cells after 4 h of conversion did not improve the reaction yield, most likely since all remaining substrate was already inside or attached to the original cells (Figure 6). However, the addition of 3 times more cells (150 g/L wet cell weight (WCW)) and 2 times more substrate (1 mM) only doubled the product concentration thereby giving a lower biocatalyst yield.

**Figure 7:**
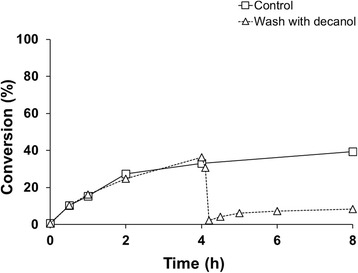
**Cell recycling by product extraction using decanol.** □ represents the control and △ the cells washed with decanol after 4 h. Reactions were performed with resting cells in shake flasks, using 1 mM cyproterone acetate and an initial catalyst concentration of 100 g/L WCW. 1 mM substrate was added when starting each reaction but only to the washed cells at 4 h.

In a further attempt to improve the biocatalyst yield, the reusability of the whole-cell system was investigated to decrease the cost contribution of the catalyst and increase the economic potential of a resting cell process. Removal of the product by solvent extraction between batches was explored. Washing with buffer did not have any effect on the product in the cell pellet fraction. Exposing the cells to the organic solvent ethyl acetate completely destroyed the activity of the cells. Furthermore, washing with decanol removed the product from the cells, but at the same time damaged the catalyst resulting in around 30% relative conversion compared to the first batch (Figure 7).

### Transport and cofactor dependence

**Figure 8:**
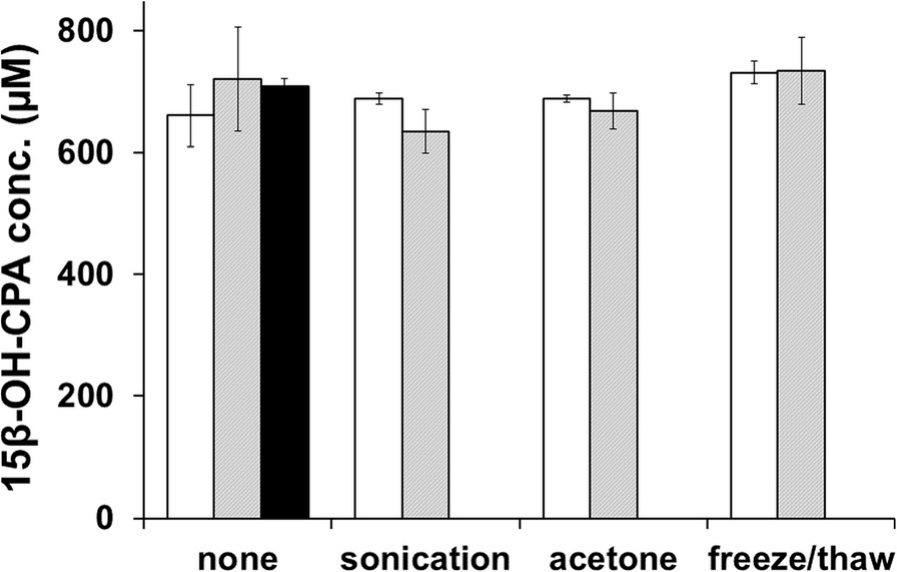
**Effect of different cell permeabilization methods and cofactor addition on 15β-hydroxycyproterone acetate production.** White bars represent the results without cofactor addition, grey bars have 1 mM NADPH and the black bar have 2 mM NADPH added. All reactions were run using 1 mM cyproterone acetate, 2% DMSO and 100 g/L WCW cells from the same fermentation batch. Error bars are 1σ.

The potential limitations of the whole-cell system in terms of substrate transport across the cell membrane and cofactor regeneration were ruled out as seen in Figure 8. The reactions were performed with resting whole-cells in shake flasks and NADPH was added in 1 mM (stoichiometric to substrate) and 2 mM final concentrations. The influence of cofactor addition and the cell permeabilization methods (sonication, acetone treatment or freeze-thawing) on the reaction rate was investigated. The initial rates measured in these experiments did not show any significant difference (data not shown). In addition, Figure 8 shows that no significant difference was observed in the final product concentration either, regardless of permeabilization treatment or external addition of cofactor. The cofactor is assumed to pass the cell membrane and enter the cell, similarily to observations in *E. coli* where this was monitored by a decreasing absorbance of the supernatant at 340 nm (unpublished work, results not shown). The results demonstrate that the cofactor regeneration of the host *B. megaterium* MS941 is sufficient to support the observed biocatalytic reaction rates and that the natural redox partners are sufficiently expressed to transport electrons from the cofactor to the active site of the overexpressed P450. The substrate transport that has been shown to limit a CYP106A2-catalyzed steroid transformation in *E. coli* [5] was not limiting the reaction studied here in *B. megaterium,* according to the tested permeabilization methods, emphasizing the suitability of this whole-cell catalyst.

### *In vivo* substrate and product inhibition

**Figure 9:**
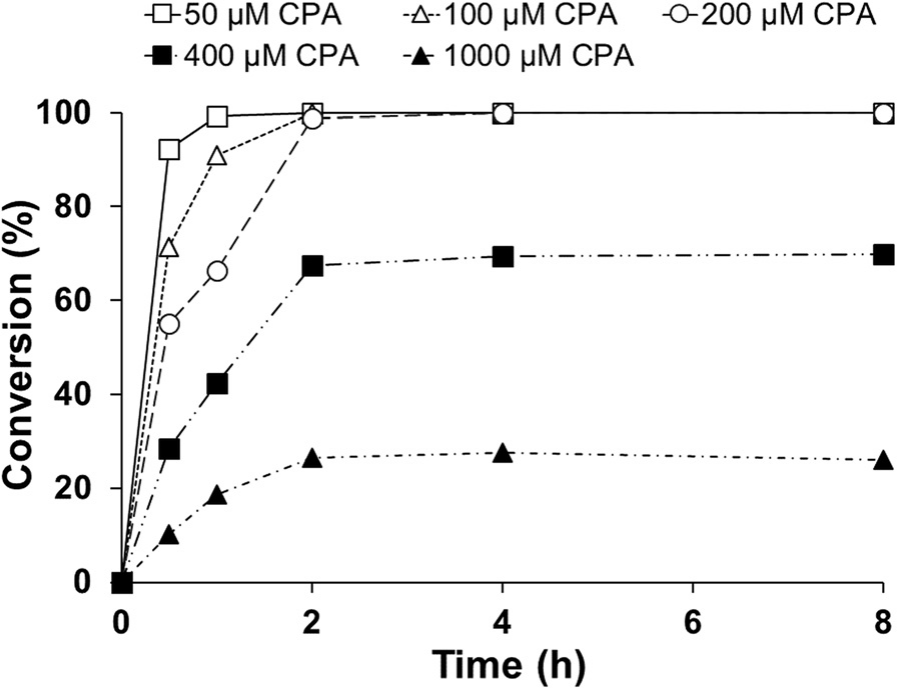
***In vivo*** **conversions performed in shake flasks with increasing substrate concentrations using resting cells.** The reactions were performed in 100 mM potassium phosphate buffer (pH 7.4) at 30°C, 150 rpm, with a substrate concentration ranging from 50 μM to 1 mM.

The influence of increasing substrate and product concentrations on the reaction performance was also investigated *in vivo* with the recombinant *B. megaterium* MS941 strain overexpressing CYP106A2. The substrate inhibition studies were performed within a concentration range of 50 μM to 1 mM. When using up to 200 μM substrate concentration, complete conversion of the substrate took place already within 2 h, while at higher substrate concentrations the conversion stopped at an approximate product concentration of 300 μM (70% conversion in the case of 400 μM and 27% in the case of 1000 μM CPA) (Figure 9), showing a similar trend as the *in vitro* experiments.

### Product removal strategies

To remove the product and push the reaction equilibrium in the forward direction, thereby enhancing the reaction performance, the use of two water-immiscible solvents, diisononylphtalate (C_26_H_42_O_4_) and hexadecane (C_16_H_34_) (selected to be compatible with the whole-cell catalyst and with oxygen requiring reactions) was investigated. However, solubilization of the substrate in these solvents, prior to addition to the aqueous phase containing the whole-cell catalyst, did not improve the reaction performance. The hydrophobicity of the substrate hinders the partitioning to the aqueous phase and thereby hampers the catalytic reaction (results not shown). The negative results could also be explained by analytical difficulties and problems with homogenous sampling in a solid–liquid 2-phase system.

Another approach to maintain a low concentration of the dissolved product in the aqueous phase is to avoid the use of a miscible co-solvent and instead apply solid substrate. This approach requires that the rate of dissolution of the substrate into the aqueous phase is faster than the reaction rate so that it does not limit the observed reaction. However, this method was not successful either, most likely due to the low solubility and rate of dissolution of the substrate.

**Figure 10:**
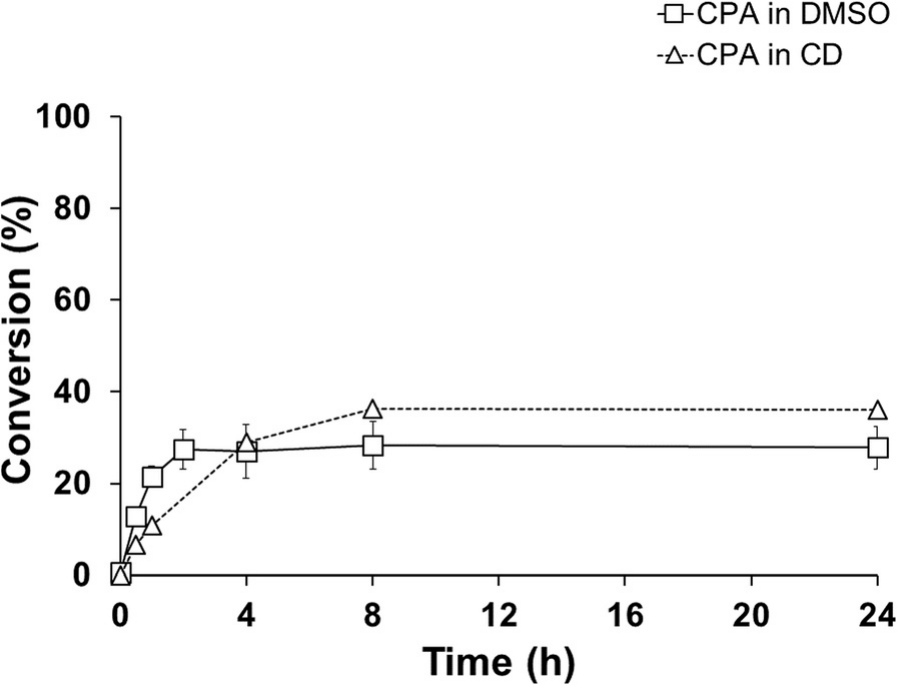
***In vivo*** **cyproterone acetate conversion in shake flasks, using 2-hydroxypropyl-** **β** **-cyclodextrin and DMSO for substrate solubilization.** The reactions were carried out with resting cells, in 100 mM potassium phosphate buffer (pH 7.4) at 30°C, 150 rpm. The substrate was pre-dissolved either in a 45% CD solution mixed overnight (△) or in DMSO (□) with a final concentration of 1 mM.

**Figure 11:**
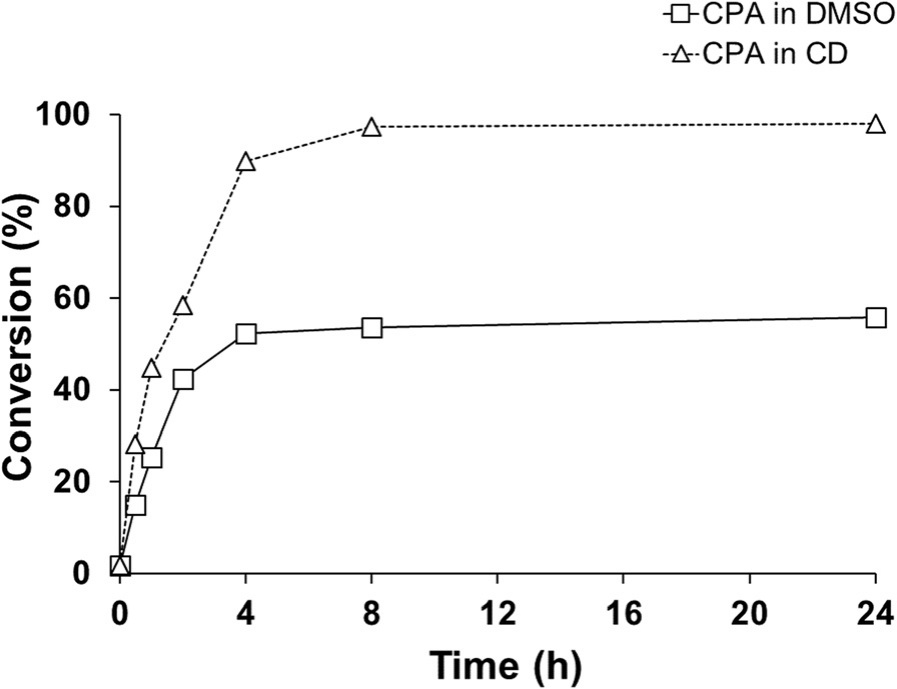
***In vivo*** **cyproterone acetate conversion in bioreactor, using 2-hydroxypropyl-β-cyclodextrin and DMSO for substrate solubilization.** The reactions were carried out in 400 mL final volume with resting cells resuspended in 100 mM potassium phosphate buffer (pH 7.4) at 30°C. The substrate was either pre-dissolved, in a 45% CD solution mixed overnight (△) or in DMSO (□) with a final concentration of 1 mM.

A third approach was to pre-solubilize the substrate in an aqueous solution of HP-β-CD ((C_6_H_9_O_5_)_7_(C_3_H_7_O)_4.5_), in order to take advantage of its multiple effects. Cyclodextrin (CD), especially the derivatized forms, have been shown to be useful in enhancing steroid conversions by e.g. increasing the cell-wall permeability, stimulating cell growth and efficiently solubilizing hydrophobic substrates [2]. The complexation of β-CD with substrate and/or product also leads to lower amounts of free dissolved species and thereby lower inhibitory effects of either substrate or product on the catalyst, as suggested previously for steroid biotransformations [23,24]. Using CD-solubilized CPA, the transformations were performed first in shake flasks with 1 mM final concentration of the substrate. As a control, the conversion was also performed with the substrate dissolved in dimethyl sulfoxide (DMSO). The CPA was added from the 45% CD solution, not exceeding 5% of the reaction volume. During the conversion, 250 μl reaction samples were taken at the indicated time points and the product/substrate ratio was analyzed by HPLC. Despite the slow initial rate, a higher conversion was reached within 4 h using CD as solubilizing agent, compared to the control. After 24 h, the conversion with CD showed 38 ± 0.05% product formation, while the control could only reach 27 ± 4.6% (Figure 10). Given the improved conversion, the same strategy was applied in the bioreactor (Figure 11). 98% conversion of 1 mM substrate was achieved on a 400 mL scale compared to a final conversion of 55% for the control without CD.

### Influence of reaction mixing

**Figure 12:**
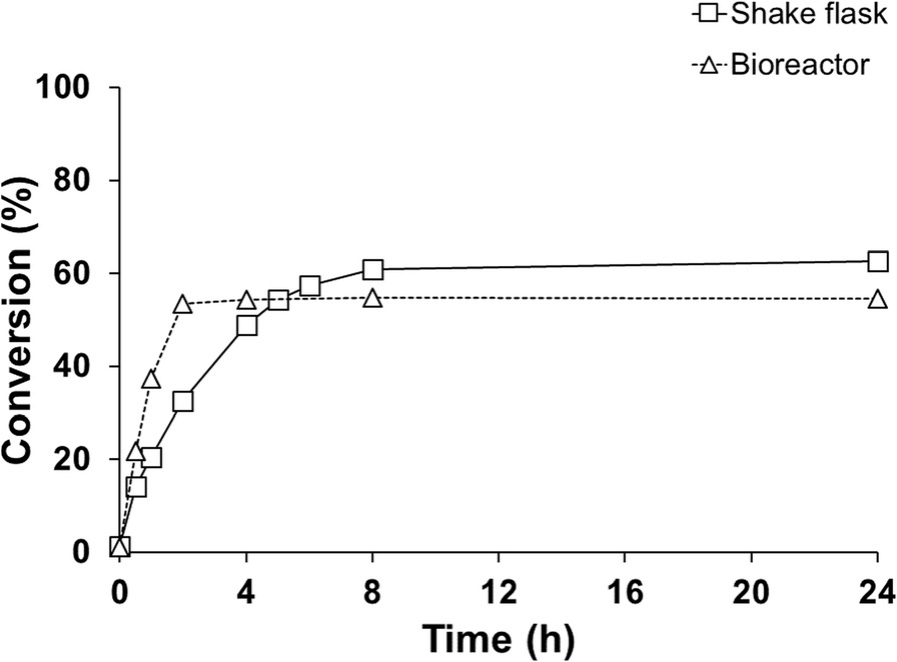
**Comparison of 1 mM cyproterone acetate transformation in bioreactor and shake flask.** The reactions were carried out using the same batch of cells, in a resting cell format (100 g/L WCW), and the substrate was added dissolved in DMSO. The initial rate of the reaction performed in the bioreactor (△) showed a higher initial rate compared to the reaction performed in shake flasks (□), although the reaction in the shake flask resulted in a higher final conversion.

In this study, catalyst-dependent parameters, believed to be independent of the used scale, were investigated in shake flasks. Shake flask experiments are commonly used in research laboratories, providing a simple and fast tool to demonstrate the proof of concept. However, for process development and scale-up studies, a more controlled environment and a more easily scalable configuration is preferred. When comparing batch transformations between bioreactor and shake flasks under identical conditions, the former showed faster initial rates (Figure 12). This result indicates that increased mixing enhances the reaction rate, most likely due to increased mass transfer of poorly water-soluble substrate but also due to the increased aeration. These results also suggest that for processes targeting scale-up and industrial implementation, process development should be performed with the intended final reactor configuration, in this case a stirred tank reactor instead of shake flasks. This especially concerns reactions involving species with low water solubility and gaseous components (e.g. oxygen).

### Catalyst stability

**Figure 13:**
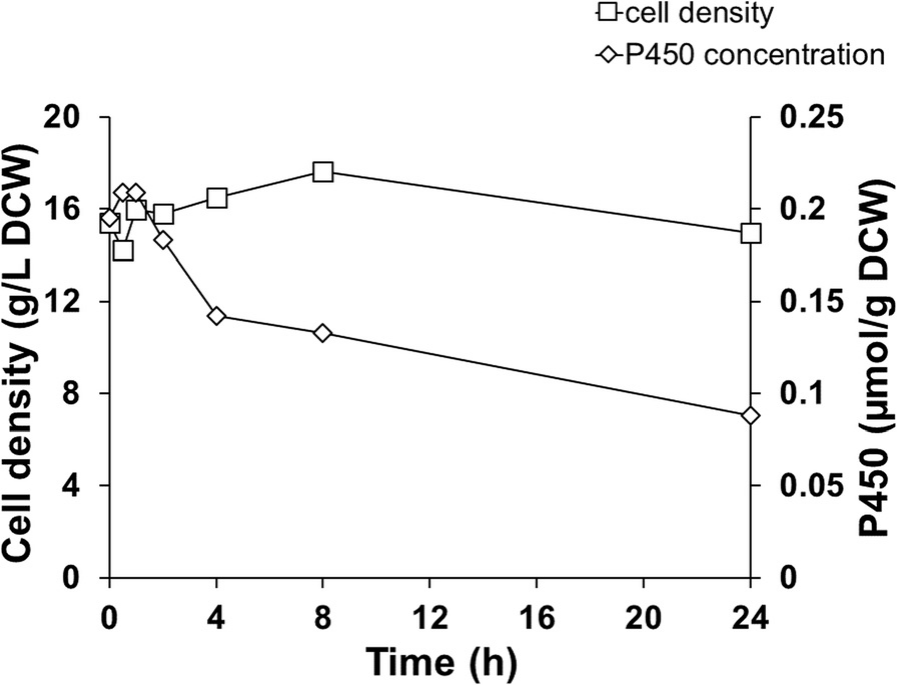
**Stability of the whole-cell catalyst under process conditions.** Reaction performed with resting cells in the bioreactor at 1 mM cyproterone acetate concentration and a cell density of 100 g/L WCW. Samples were collected at the indicated time points. The dry cell weight (□) and the P450 content (◇) were measured.

The stability of a biocatalyst is crucial for the economic potential of a biocatalytic process. In this study we examined the storage stability, due to practical reasons, and more importantly, the stability under process conditions, under which the relevant information is collected. The dry cell weight and the correctly folded P450 were monitored during the process and it was found that the cells remained intact but the stability of the CYP106A2 was limited. One third of the correctly folded P450 is degraded after 4 h of the reaction and more than 50% is lost after 24 h (Figure 13).

The storage stability of the catalyst was determined by running resting cell biotransformations with cells previously resuspended in buffer and stored gently shaken at 4°C. No significant loss in reaction performance was found over 7 days of storage (data not shown). The stability of the catalyst could potentially also be affected by the mode of operation. However, no significant difference was seen between growing and resting cells in terms of conversion, whole-cell stability or P450 stability.

## Discussion

Even though in the past decade significant progress was made to develop efficient biocatalysts for steroid transformations, there is still a great demand for greener and cost-efficient routes for valuable steroid production [2]. Microbial steroid transformations, using heterologously expressed cytochrome P450 enzymes are considered to be a promising approach, since the regio- and stereospecific hydroxylations are known to be challenging for synthetic steroid chemistry, suffering from low predictability and specificity [1,25]. However, in most cases the reactions performed at analytical scale are not directly applicable for industrial practice. For an industrial process, the economic feasibility needs to be considered. For guidance, at the current stage of process development, targets for economic metrics such as space-time yield (for a growing cell process), final product concentration and biocatalyst yield (for a resting cell process) can be used. For high-value products or pharmaceuticals, such as the 15β-OH-CPA, target values for product concentration of 20 g/L, a space-time yield of 2 g/L/h (for a growing cell process) and a biocatalyst yield of 10 g product/g dry cell weight (DCW) (for a resting-cell process) can be used as reference [21]. These targets can only be achieved using higher substrate concentrations, and problems associated with higher concentrations such as low solubility of the substrate, potential substrate or product inhibition and toxicity most likely will arise.

In a previous study, Hannemann and co-workers converted 11-deoxycorticosterone to 15β-hydroxy-11-deoxycorticosterone using CYP106A2, functionally expressed with bovine adrenal redox partners in a growing whole-cell *E. coli* host. According to our calculations, the molar substrate concentration applied in this study (0.5 mM) could result in a maximal final product concentration of 0.17 g/L. The space-time yield was reported to be 0.33 g/L/d, however, it has to be taken into account that the substrate was not fully converted after 24 h [13]. Nevertheless, the focus of the study was to design a whole-cell catalyst and demonstrate the applicability of a screening assay. The whole-cell system was then further improved by the coexpression of a cofactor regeneration system and was used for the 15β-hydroxylation of progesterone and testosterone [5]. This system was shown to be limited by substrate transport across the cell membrane and solubility of the substrate was shown to be crucial for the reaction performance. The productivity concerning testosterone hydroxylation was reported to be 5.5 g/L/d using 0.5 mM initial substrate concentration and a lyophilized cell extract on a 20 mL scale, however, this value was extrapolated from a 30 min reaction. Taking into account the 0.5 mM initial substrate concentration the 96% conversion could only lead to a final product concentration of 0.15 g/L. In the same study experiments with growing cells showed only 25% selectivity towards the 15β position, considerably lower than resting cells (≥80%). More recently, DHEA was regioselectively hydroxylated to 7β-OH-DHEA by CYP106A2 expressed in *B. megaterium* [12]. A benchmark 0.44 g/L total final product concentration was obtained upon addition of 1.5 mM substrate in a repeated batch mode, yielding 6 different products in 12 h, which is the highest reported concentration of the studies published so far. The selectivity towards the 7β-position was improved from 0.7 to 0.9 by changing the host from *B. megaterium* strain ATCC 13368 to MS941. The bottlenecks of the reaction were discussed but never investigated.

In our study a systematic approach was taken to identify catalyst- and reaction-related limitations to enable an efficient process with defined targets based on economic guidance. By addressing the identified limitations we managed to evaluate and improve the process performance. This is a novel approach compared to previous studies on steroid hydroxylation by CYP106A2. We investigated the regio- and stereoselective 15β-oxidation of CPA, in shake flasks and laboratory scale bioreactors. CYP106A2 was overexpressed in *B. megaterium* MS941 and proved to be a robust catalyst for the synthetic biotransformation of CPA. The *B. megaterium* host offers a protected environment for the enzyme, enhancing its stability, besides the cofactor regeneration provided by the cellular metabolism, which, according to our studies, was shown to be sufficient to not limit catalysis. The substrate transport across the membrane of the *B. megaterium* whole-cell catalyst turned out not to be a limiting factor either, as was described in the case of steroid hydroxylation by *E. coli* [5]. In contrast, the low overall solubility of the reactants, the limited stability of the P450, in combination with product inhibition, are suggested to be the main bottlenecks of this system.

The low solubility of substrate is a common challenge to many P450-catalyzed reactions. In the case of CPA the solubility is reported to be 0.64 mg/L [26] and 34 mg/L for the hydroxylated product [27]. However, this should be put in perspective to the aimed final product concentration of 20 g/L. Low steroid solubility is usually solved by the application of a water immiscible co-solvent but this approach has limitations at higher concentrations when the co-solvent can damage the biocatalyst [28]. This can partly be circumvented by the application of CDs. These cyclic oligosaccharides are used in e.g. drug delivery [29] and have also been applied in P450-catalyzed reactions, mainly as a solubilizing agent and to improve substrate transport across the cell membrane, however at limited substrate concentrations (50 mg/L) [23]. The effects of HP-β-CD on steroid dehydrogenation of the gram-positive bacteria *Arthrobacter simplex* was investigated by Shen and coworkers [30]. Cells pretreated with HP-β-CD showed double the initial rate and reached final conversion 1 h faster compared to the non-treated cells, although in the end both reached the same concentration. This was shown to be the result of the cell membrane permeabilization by altered lipid and protein profiles of the membrane. CDs have also been applied in other fermentation and biotransformation processes to avoid toxic and inhibitory effects of the substrate or product, as summarized by Singh *et al.* [31]. In this work, using HP-β-CD, we were able to achieve 98% conversion of 1 mM CPA in a regio- and stereoselective manner within 8 h, resulting in 0.43 g/L product. This matches the literature benchmark for steroid conversion by CYP106A2 [12], yet the 8 h conversion time applied here improves the space-time yield by 31%. This new approach of bottleneck identification, takes P450-catalyzed reactions one step further towards higher product titers and economic viability. In our system, the time-dependent product inhibition was shown to limit final achievable product concentrations at higher substrate concentrations, thereby decreasing the reaction yield. By overcoming this problem, the main effect of CD was believed to be the complexation of the product in the lipophilic central cavity, thereby pulling it out of the hydrophilic environment present in the cell and pushing the equilibrium towards the product formation.

## Conclusion

Ultimately, with the help of HP-β-CD, we could achieve a nearly complete conversion and a product formation of 0.43 g/L at a 400 mL scale, getting closer to industrial process requirements and a future large-scale application. However, in order to fully exploit the potential of the CD process, further optimization studies should be performed. Using CD to circumvent the identified bottlenecks of solubility and product inhibition, the stability of the P450 is still a challenge for an economically feasible process. Considering that the stability of the enzyme will make cell recycling difficult, and that no significant differences could be found between a growing and resting cell process, the preferred mode of operation for further process development would be growing cells. As stated above, the targets for an economically feasible growing cell process are a final product concentration of 20 g/L and a space-time yield of 2 g/L/h [21]. Although we could demonstrate a successful process development towards the suggested values by addressing the limitations, there is still room for improvement.

## Materials and methods

### Reagents and chemicals

All used chemicals were from standard sources and of highest grade available. Solvents of analytical grade were used for HPLC, while solvents used for large-scale extraction were of reagent grade. CPA was obtained from BIOTREND Chemikalien GmbH, Köln, Germany (≥98% (HPLC)).

### Bacterial strains and plasmids

The *in vivo* conversions were performed using a recombinant *B. megaterium* MS941 strain, a variant of DSM319, lacking the major extracellular protease gene *nprM* [32]. The host organism was transformed with the plasmid pSMF2.1C (CYP106A2 gene introduced within the SpeI/MluI sites) by a polyethylene glycol-mediated technique using protoplasts [9,16]. As control, the wild type *B. megaterium* MS941 strain was used (lacking the pSMF2.1 plasmid, but naturally containing cytochrome P450 genes) to confirm that the reaction is catalyzed by the CYP106A2 enzyme. The wild type strain did not display any activity towards the substrate (data not shown) and no P450 was detected using CO difference spectroscopy [33], indicating that the P450 expression and catalytic activity reported is assigned to the overexpressed CYP106A2.

### Protein expression, purification and spectral characterization

The expression and purification of the CYP106A2 protein was performed as described previously [6,34]. For the reconstituted *in vitro* system, a truncated form of bovine adrenodoxin (Adx_4–108_) was used in combination with bovine adrenodoxin-reductase (AdR), their expression and purification was completed as described elsewhere [35,36]. The characteristics of the purified CYP106A2 were analyzed by UV-visible absorbance spectroscopy. The spectrum was recorded in a range of 200 to 700 nm with a double beam spectrophotometer (UV-1800, UV-2101 PC, Shimadzu Corporation, Kyoto, Japan) and analyzed constantly during the purification process, to determine the Q value (A_417_/A_280_), which was in all cases above 1.5, suggesting a high amount of correctly folded, active P450s. The samples taken from the bacterial cultures during cultivation or conversion were spun down and the pellet kept frozen at -20°C until measurement when the samples were resuspended in 100 mM potassium phosphate buffer, pH 7.4. The concentration of the purified protein and the protein expressed in the whole-cell system was determined by CO difference spectroscopy according to the method of Omura and Sato [33], using an extinction coefficient of 91 mM^−1^ cm^−1^.

### Substrate binding assay

The substrate binding spectrum was investigated using a double-beam spectrophotometer (UV-2101PC, Shimadzu, Japan) and tandem quartz cuvettes. The analysis took place in 800 μL total volume. One chamber of each cuvette contained 10 μM solution of CYP106A2 in 50 mM potassium phosphate buffer pH 7.4, while the other chamber was filled with buffer. CPA was dissolved in DMSO at a stock concentration of 10 mM. The enzyme was titrated with the substrate in a concentration range of 5 to 150 μM. After each titration step the spectrum was recorded in a range of 350 to 500 nm.

### *In vitro* conversions and enzyme kinetics

The *in vitro* conversion of CPA was carried out with a reconstituted system in a final volume of 250 μL at 30°C for 30 min in 50 mM potassium phosphate buffer (pH 7.4), containing 20% (v/v) glycerol. The reconstituted system contained bovine AdR (1 μM), a truncated form of Adx_4–108_ (10 μM), CYP106A2 (0.5 μM), a NADPH-regenerating system [MgCl2 (1 mM), glucose-6-phosphate (5 mM), glucose-6-phosphate dehydrogenase (1 U), and NADPH (0.1 μM)], and CPA (200 μM). The reaction was started by adding NADPH (1 mM) and incubated at 30°C. The assay was stopped by the addition of 250 μL of ethyl acetate, mixed vigorously, and extracted twice. The combined organic phases were evaporated and the residues were dissolved in the HPLC mobile phase (60:40% ACN:H_2_O) and subjected to analysis.

Reaction kinetics of CPA and CYP106A2 were performed for 2 min as described above, using bovine AdR (0.5 μM), bovine Adx_4–108_ (5 μM) and CYP106A2 (0.25 μM). The substrate concentration varied from 20 to 250 μM. Product amounts were determined by plotting the amount of product formed (nmol product/nmol P450/min) against the substrate concentration (μM). Each reaction was performed six times, the data represents the mean of these independent results. Data were fitted by hyperbolic regression with the help of Origin (OriginLab Corporation, Massachusetts, USA).

The substrate inhibition studies were performed using a 20 mM CPA stock solution dissolved in DMSO and added in a final concentration of 200 to 1200 μM. Studying the product inhibition, the substrate was added in a final concentration of 400 μM, while the purified product concentration ranged from 0 to 1200 μM. The reactions took place for 60 min, then the samples were extracted and subjected to HPLC analysis as described before.

### Heterologous expression in shake flasks

Pre-cultures were inoculated from a -80°C glycerol stock, using 25 mL complex TB medium (24 g/L yeast extract, 12 g/L soytone, 2.31 g/L KH_2_PO_4_ and 12.5 g/L K_2_HPO_4_) supplemented with 10 mg/L tetracycline and incubated overnight, at 150 rpm, 30°C. The main culture, containing 250 mL complex medium (supplemented with the corresponding amount of tetracycline) was inoculated with 1% of the culture volume from the pre-culture. The main culture was incubated until an OD_578_ = 0.5, when 5 g/L xylose solution was added to induce expression. After 24 h expression, the cells were harvested (4500 x g, 4°C, 15 min), the cell pellet was washed and resuspended in 100 mM potassium phosphate buffer (pH 7.4).

### Heterologous expression at fermenter scale

The fermentation process was adapted from Korneli and coworkers [37]. A -80°C glycerol stock was used to inoculate the first pre-culture with LB medium which was used to inoculate a second pre-culture with batch medium (3.52 g/L KH_2_PO_4_; 6.62 g/L Na_2_HPO_4_*2H_2_O; 0.3 g/L MgSO_4_*7 H_2_O; 25 g/L (NH_4_)2SO_4_; 1 g/L yeast extract and trace elements as described [37]). The first pre-culture was incubated for 8 h in a 100 mL shake flask with 10 mL LB medium at 150 rpm, 37°C. 100 mL batch medium supplemented with 5 g/L fructose was used for the cultivation of the second pre-culture in a 1 L flask, inoculated from the first pre-culture to an OD_600_ of 0.1. After 12 h of cultivation, it served as inoculum for the fermenter. The batch medium in the fermenter was supplemented with 15 g/L fructose. The feed medium consisted of 150 g/L fructose; 5 g/L D-xylose; 9.9 g/L KH_2_PO_4_; 14.98 g/L Na_2_HPO_4_; 0.3 g/L MgSO_4_*7H_2_O, 25 g/L (NH_4_)_2_SO_4_. All media preparations were supplemented with 10 mg/L tetracycline. The fed-batch fermentation process was carried out in a 1 L Infors Multifors fermentation vessel (Infors HT, Bottmingen, Switzerland). Initial conditions were set to 37°C; pH 7.0; aeration 0.5 Lpm and pO_2_ setpoint 20% controlled by stirrer. The fermenter was inoculated to a final OD_600_ of 0.7 and induced at an OD_600_ of 10 with 5 g/L xylose. The induction at a higher OD_600_ in the fermenter, compared to shake flask, is due to the higher possible final cell density utilizing a fed-batch process. At the time of induction, the temperature was decreased to 30°C, agitation was increased to 1 Lpm and a linear feed was initiated. The pH was controlled during the process with 5 M NaOH and 1 M H_3_PO_4_. Cells were harvested by centrifugation and washed by resuspension in 100 mM potassium phosphate buffer, pH 7.4. 20 mM fructose was added to the cells for direct use in biotransformation or stored at 4°C, gently shaking, at a cell density of 200 g/L WCW.

### Bioconversion in shake flasks

The small scale conversion of CPA was performed with resting cells in a 15 mL culture volume using 100 mL baffled shake flasks. The catalyst concentration was 100 g/L WCW, unless otherwise stated. To obtain sufficient amount of product (mg) for NMR structure characterization, the conversion was scaled up to 50 mL, using 300 mL baffled shake flasks. CPA was added in 200 μM final concentration from a 20 mM DMSO stock solution. The use of DMSO did not exceed 2% of the culture volume. Every 4 h, 2 M fructose solution was added as carbon source in a final concentration of 20 mM. 250 μL samples were taken at indicated time points to monitor the conversion. The samples were extracted twice using 250 μL ethyl acetate, the organic phases collected and evaporated to dryness for the subsequent analysis by reverse-phase HPLC. In the case of product isolation, the reaction was stopped and the steroids were extracted twice by ethyl acetate. The organic phase was dried over anhydrous MgSO_4_ and concentrated to dryness in a rotavapor (Büchi R-114). The yellowish residue was dissolved in the mobile phase of the HPLC and filtered through a sterile syringe filter (Rotilabo syringe filter, 0.22 μm, Carl Roth GmbH, Karlsruhe, Germany). The product purification was completed by preparative HPLC, according to its retention time. The collected fractions were evaporated to dryness and analyzed by NMR spectroscopy. The *in vivo* substrate and product inhibition was studied in shake flasks, using resting cells, as described for the *in vitro* studies.

### HPLC analysis

The HPLC analysis was performed either on a Jasco system consisting of a Pu-980 HPLC pump, an AS-950 sampler, a UV-975 UV/Vis detector, and an LG-980–02 gradient unit (Jasco, Gross-Umstadt, Germany) or on a Dionex UltiMate 3000 HPLC equipped with a Photodiode Array Detector (Dionex, Thermo Scientific, Sunnyvale, CA, USA). A reversed-phase ec MN Nucleodor C_18_ (3 μM, 4.0x125 mm) column (Macherey-Nagel, Betlehem, PA, USA) was used and kept at an oven temperature of 40°C. An isocratic gradient of acetonitrile:water in a ratio of 60:40 was applied using a flow rate of 0.8 mL/min. UV detection of the substrate and product was accomplished at 282 nm. Product isolation was performed using preparative reversed-phase HPLC (ec MN Nucleodur C18 VP (5 μM, 8x250 mm), Macherey-Nagel, Betlehem, PA, USA) with a flow rate of 2.5 mL/min. The results are presented as conversion %, calculated from the product area divided by the sum of substrate and product areas. Regarding the product inhibition experiments, when product was added initially to the system, the data is presented as relative conversion (subtracting the initially added product) or calculated back to concentrations from conversions.

### NMR characterization of the main metabolite

The NMR spectrum was recorded in CDCl_3_ with a Bruker DRX 500 NMR spectrometer at 300 K. The chemical shifts were relative to TMS using the standard δ notation in parts per million. The 1D NMR (^1^H and ^13^C NMR, DEPT135) and the 2D NMR spectra (gs-HH-COSY, gs-NOESY, gs-HSQCED, and gs-HMBC) were recorded using the BRUKER pulse program library. All assignments were based on extensive NMR spectral evidence.

The product (P1) was identified as 15β-hydroxy cyproterone acetate (15β-OH-CPA) (3.4 mg). In comparison to cyproterone acetate the NMR spectra of its conversion product showed signals for an additional secondary hydroxyl group (δ_H_ 4.50 ddd, J = 7.5, 6.0 and 2.0 Hz; δ_C_ 68.86). Its position at C-15 could be deduced by vicinal couplings of the methin proton with H-14 (δ_H_ 1.93 dd, J = 12.0 and 6.0 Hz), H-16α (δ_H_ 2.47 dd, J = 16.8 and 7.5 Hz) and H-16β (δ_H_ 3.03 dd, J = 16.8 and 2.0 Hz) in the HHCOSY and with C-13 (δ_C_ 47.00) and C-17 (δ_C_ 96.11) in HMBC. The β orientation of the hydroxyl was obvious by the NOESY effects of H-15 to H-16α and to H-9 (δ_H_ 1.53 m) and H-14, both in α position. In addition, the coupling constants found for H-15α resembled those for other closely related steroids, e.g. 15β-hydroxy-11-deoxycortisol [8]. Selected ^1^H NMR data of 15β-hydroxy cyproterone acetate could be found in the literature [38] and matched with our values. ^1^H NMR (CDCl_3_, 500 MHz): δ 0.89 dt (6.3 and 4.7 Hz, cPr-Ha), 1.01 s (3xH-18), 1.27 s (3xH-19), 1.29 ddd (9.0, 7.8 and 4.7 Hz, cPr-Hb), 1.53 m (H-9), 1.62 m (2H, H-11β and H-12β), 1.74 td (7.8 and 6.3 Hz, H-1), 1.93 dd (12.0 and 6.0 Hz, H-14), 1.98 m (H-11α), 2.03 m (H-2), 2.04 m (H-1 2α), 2.09 s (3x OCOC*H*_3_), 2.10 s (3xH-21), 2.47 dd (16.8 and 7.5 Hz, H-16α), 2.73 td (12.0 and 2.3 Hz, H-8), 3.03 dd (16.8 and 2.0 Hz, H-16β), 4.50 ddd (7.5, 6.0 and 2.0 Hz, H-15), 6.20 brs (H-4), 6.44 d (2.3 Hz, H-7). ^13^C NMR (CDCl_3_, 125 MHz): δ 12.30 (CH_2_, cPr), 16.95 (CH_3_, C-18), 20.74 (CH_2_, C-11), 21.11 (C, OCO*C*H_3_), 22.86 (CH_3_, C-19), 25.24 (CH, C-2), 26.12 (CH, C-1), 26.35 (CH_3_, C-21), 31.96 (CH_2_, C-12), 34.44 (CH, C-8), 38.84 (C, C-10), 43.13 (CH_2_, C-16), 47.00 (C, C-13), 47.98 (CH, C-9), 52.80 (CH, C-14), 68.86 (CH, C-15), 96.11 (C, C-17), 120.47 (CH, C-4), 130.45 (C, C-6), 136.30 (CH, C-7), 152.32 (C, C-5), 170.51 (C, O*C*OCH_3_), 197.98 (C, C-3), 202.84 (C, C-20).

### Transport and cofactor dependence

Transport limitation was examined by different cell membrane permeabilization methods prior to the biocatalytic reaction. Both mechanical and chemical methods were applied (freeze-thawing, acetone treatment and ultra-sonication). Frozen cells were spun down and the pellet was kept at −20°C overnight. Acetone-treated cells were incubated with 5% acetone for 2 min while vortexing and mechanical disruption was performed by sonication for 2 min (amplitude 60%, 0.5 s cycles) (UP400 S; Hielscher Ultrasonic GmbH, Teltow, Germany). Following the permeabilization treatment (acetone and sonication) cells were spun down and the pellet resuspended in 100 mM potassium phosphate buffer pH 7.4. Cofactor was added to the untreated control samples and to the permeabilized ones, once and twice stoichiometric amounts relative to substrate concentration.

### Storage stability

To examine the storage stability of the whole-cell catalyst, the transformation was performed with resting cells after 1, 3 and 7 days of storage. The cells were stored at 4°C, gently shaking at a cell density of 200 g/L WCW in 100 mM potassium phosphate buffer, pH 7.4. 20 mM fructose was added as carbon source at the time of harvest, after 1 and 3 days of storage and also at the start of the reactions.

### Cyclodextrin

As an alternative substrate feeding strategy, the substrate was pre-dissolved in a 45% (w/v) solution of HP-β-CD in sterile-filtered MilliQ water and stirred overnight using a magnetic stirrer.

### Optical density and dry cell weight determination

To estimate the cell concentration, the optical density at 600 nm (OD_600_) was monitored and the gravimetric dry cell weight (g/L DCW) was determined. Samples collected for dry cell weight measurement were spun down, the supernatant was discarded and the pellet was kept at −20°C until further analysis. Thereafter the pellets were thawed and resuspended in the original sample volume using 100 mM potassium phosphate buffer, pH 7.4. Dry cell weight was measured in triplicates by filtering the samples through a pre-weighed 0.22 μm PES membrane filter (Frisenette, Knebel, Denmark) applying vacuum. The filters were washed with buffer, dried in a microwave oven and weighed after equilibrating to room temperature in a desiccator.

### Bioconversion in bioreactor

Biocatalysis in bioreactors was performed in the same vessels as the fermentation (Infors Multifors, Infors HT, Bottmingen, Switzerland) in a working volume of 400 mL. Set points applied were: 30°C, aeration 1 Lpm, pO_2_ 30% controlled by agitation, pH 7.2 controlled with 5 M NaOH and 1 M H_3_PO_4_. 2 M stock solution of fructose was added at time point 0, 4 and 8 h in a final concentration of 20 mM. CPA was dissolved in DMSO and added in a final concentration of 1 mM, the DMSO content not exceeding 2% of the total volume. For an accurate comparison of growing and resting cells half of the fermentation volume was removed after 16 h and this fraction was harvested by centrifugation, washed and resuspended in 100 mM potassium phosphate buffer, pH 7.4. Resuspended cells were transferred to a bioreactor, parallel to the still growing cells. Fructose and xylose were continuously fed, for both resting and growing cells, at half the volumetric rate compared to the fermentation due to half the volume.

## Abbreviations

AdR: Adrenodoxin reductase
Adx: Adrenodoxin
*B. megaterium*: *Bacillus megaterium*
CD: Cyclodextrin
CPA: Cyproterone acetate, 15β-OH-CPA, 15β-hydroxycyproterone acetate
DHEA: Dehydroepiandrosterone
DMSO: Dimethyl sulfoxide
DCW: Dry cell weight
HP-β-CD: 2-hydroxypropyl-β-cyclodextrin
HPLC: High-performance liquid chromatography
*E. coli*: *Escherichia coli*
NMR: Nuclear magnetic resonance
P450: Cytochrome P450 enzymes
WCW: Wet cell weight
